# Comprehensive genetic testing for female and male infertility using next-generation sequencing

**DOI:** 10.1007/s10815-018-1204-7

**Published:** 2018-05-19

**Authors:** Bonny Patel, Sasha Parets, Matthew Akana, Gregory Kellogg, Michael Jansen, Chihyu Chang, Ying Cai, Rebecca Fox, Mohammad Niknazar, Roman Shraga, Colby Hunter, Andrew Pollock, Robert Wisotzkey, Malgorzata Jaremko, Alex Bisignano, Oscar Puig

**Affiliations:** Phosphorus, Inc., 1140 Broadway St, New York, NY 10001 USA

**Keywords:** Infertility, Next-generation sequencing, Clinical genetic testing, Diagnostic

## Abstract

**Purpose:**

To develop a comprehensive genetic test for female and male infertility in support of medical decisions during assisted reproductive technology (ART) protocols.

**Methods:**

We developed a next-generation sequencing (NGS) gene panel consisting of 87 genes including promoters, 5′ and 3′ untranslated regions, exons, and selected introns. In addition, sex chromosome aneuploidies and Y chromosome microdeletions were analyzed concomitantly using the same panel.

**Results:**

The NGS panel was analytically validated by retrospective analysis of 118 genomic DNA samples with known variants in loci representative of female and male infertility. Our results showed analytical accuracy of > 99%, with > 98% sensitivity for single-nucleotide variants (SNVs) and > 91% sensitivity for insertions/deletions (indels). Clinical sensitivity was assessed with samples containing variants representative of male and female infertility, and it was 100% for SNVs/indels, *CFTR* IVS8-5T variants, sex chromosome aneuploidies, and copy number variants (CNVs) and > 93% for Y chromosome microdeletions. Cost analysis shows potential savings when comparing this single NGS assay with the standard approach, which includes multiple assays.

**Conclusions:**

A single, comprehensive, NGS panel can simplify the ordering process for healthcare providers, reduce turnaround time, and lower the overall cost of testing for genetic assessment of infertility in females and males, while maintaining accuracy.

**Electronic supplementary material:**

The online version of this article (10.1007/s10815-018-1204-7) contains supplementary material, which is available to authorized users.

## Introduction

It is estimated that 48 million couples were affected by infertility in 2010, and there has not been any significant improvement in infertility levels between 1990 and 2010 [1–3]. In the USA, 12% of women aged 15–44 have impaired fecundity. Increasingly, more couples rely on assisted reproductive technologies (ART) to get pregnant and have children, and in the USA, 231,936 ART cycles were performed in 2015 [4].

A significant proportion of infertility cases are due to genetic defects. Male infertility accounts for 50% of infertility cases [5], with known genetic factors accounting for 15–30% of male infertility [6]. Chromosomal alterations [7], inversions [8], translocations [9], Y chromosome microdeletions [10], and gene mutations (for example single-nucleotide variants (SNVs) in *CFTR* [11]) are the main genetic variants causing male infertility. In females, infertility is a more heterogeneous condition. While genetics clearly play a role, these effects are mostly polygenic, making it difficult to define a single genetic cause. The two most common female factor conditions, ovulatory dysfunction (25%) and endometriosis (15%), have familial predisposition, suggesting a genetic basis [12]. In addition, sex chromosome alterations [13] and several single gene mutations have been described impacting female fertility [14, 15], causing conditions like hypogonadotropic hypogonadism, premature ovarian insufficiency, endometriosis, and polycystic ovarian syndrome (reviewed in [12]).

Traditionally, several assays are needed to make a definitive genetic diagnosis of infertility, which makes the process expensive and slow. For example, in males, a variety of techniques are necessary for analysis of genetics: sex chromosome aneuploidies are detected by cytogenetic tests like karyotyping; Y chromosome microdeletions are detected by polymerase chain reaction (PCR)-based methods; and *CFTR* mutations are detected by Sanger DNA sequencing. However, a shotgun approach in which all genetic tests are ordered for all patients is not recommended because cost is prohibitive [16]. In females, success rates vary depending on many factors with age being the most important. An infertility evaluation includes very diverse tests including blood and urine hormone levels, imaging, and for women with unexplained infertility issues, genetic tests like karyotyping or DNA sequencing of selected genes.

Next-generation sequencing (NGS) permits the simultaneous interrogation of multiple disease-causing variants in many genes, allowing expanded genetic diagnostics to be routinely used in medical practice. This is already a reality in other medical fields, like oncology [17] and heart disease [18], where panel testing allows for the most comprehensive assessment of genetic etiologies. NGS is also very cost-effective as it allows for the detection of very different types of variants (for example, SNVs, small indels, large Y chromosome deletions, and sex chromosome aneuploidies) by using a single test in combination with multiple bioinformatics algorithms to process these diverse data. Here, we present the development of an NGS panel and bioinformatics pipeline for the detection of genetic variants with direct impact on female and male infertility. We also present a cost comparative analysis of current approaches in comparison with the NGS test.

## Materials and methods

### Subjects and samples

Samples of genomic DNA with previously identified variants in the genes interrogated by the sequencing panel were obtained as purified DNA from the Human Genetic Cell Repository (National Institute of General Medical Sciences) at the Coriell Institute for Medical Research (Camden, NJ) or the Indiana Biobank (Indianapolis, IN) or extracted from saliva samples collected by DNAsimple (Philadelphia, PA). All samples submitted to our diagnostics laboratory as saliva were manually extracted using the Qiagen QIAamp Mini Kits following the manufacturer’s instructions. DNA was quantified using Quant-iT Picogreen dsDNA Assay Kit (Life Sciences) and a Varioskan LUX (Thermo Scientific). Supplementary Table S1 describes all samples used in this study, with biorepository numbers, as well as their previous characterization results and rationale for inclusion. All samples presented here are de-identified (HHS 45 CFR part 46.101(b)(4)). IRB approval to handle de-identified samples was obtained through ASPIRE (Santee, CA), protocol IRB-R-003.

### Fertility panel description

A targeted next-generation sequencing panel consisting of 87 genes related to infertility disorders was created (Supplementary Table S2). Genes relevant to disease phenotype were included based on relationships described in Online Mendelian Inheritance in Man (OMIM), Human Phenotype Ontology (HPO), GeneReviews, and primary literature. The panel included all coding exons, splice sites, promoter regions, 5′ untranslated regions (UTRs), and 3′ UTRs for each of the genes. Clinically relevant noncoding (intronic) regions that contained previously described pathogenic variants, as reported in ClinVar NIH database, were also included. The total size of the gene panel is 1,444,982 bp. In addition, selected regions from the Y chromosome (928,649 bp) were included to allow for the detection of Y chromosome microdeletions. Finally, the panel also includes 188 genes that we considered “research” genes, with links to infertility and which we wanted to follow for research purposes (Supplementary Table S3).

### Next-generation DNA sequencing

All laboratory procedures were performed in a Clinical Laboratory Improvement Amendments (CLIA) laboratory. DNA samples were prepared for sequencing using HyperPlus Library Preparation Kit (Roche, Indianapolis, IN) and sequenced on a NextSeq500 (Illumina, San Diego, CA), following the manufacturer’s instructions. A detailed sequencing protocol is provided in Supplementary Methods section.

### Variant identification and classification

All bioinformatics algorithms were implemented within the Elements^™^ platform (Phosphorus, New York, NY). FASTQ files were produced from each sequencing run and processed using the germline calling pipeline (version 2.03.01.30066) in DRAGEN (Edico Genome, San Diego, CA). Variants identified by NGS were confirmed by an orthogonal method (microarrays or Sanger sequencing). After confirmation, each variant was classified as pathogenic, likely pathogenic, variant of unknown significance (VUS), likely benign, or benign, following the American College of Medical Genetics (ACMG) guidelines [19].

### Orthogonal confirmation

A custom Affymetrix Axiom array was used to confirm all the reportable variants including SNVs, indels, CNVs, Y chromosome microdeletions, and sex chromosome aneuploidies. Microarrays were processed following the manufacturer’s instructions. To confirm variants not included in the microarray, Sanger sequencing was used. Orthogonal analysis was also performed for cases when there was discrepancy between expected and received NGS results. If both NGS and confirmatory results agreed, the results were counted as concordant. Detailed protocols are described in the Supplementary Methods section.

### FMR1 testing

*FMR1* testing was performed with 120 ng genomic DNA per sample and the AmplideX PCR CE *FMR1* kit (Asuragen, Austin, TX), following the manufacturer’s instructions. PCR products were separated in a 3500XL capillary electrophoresis system (Applied Biosystems, Foster City, CA) using conditions described in the *FMR1* kit manual.

## Results

### Gene panel design

**Fig. 1:**
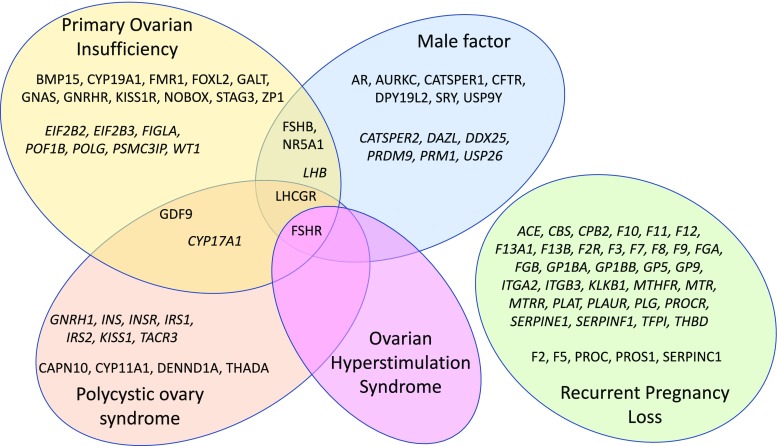
Description of the NGS panel by gene content, organized by the main infertility indications. Genes classified as “diagnostic” are shown in standard font, and genes classified as “informative” are shown in *italic font*

**Fig. 2:**
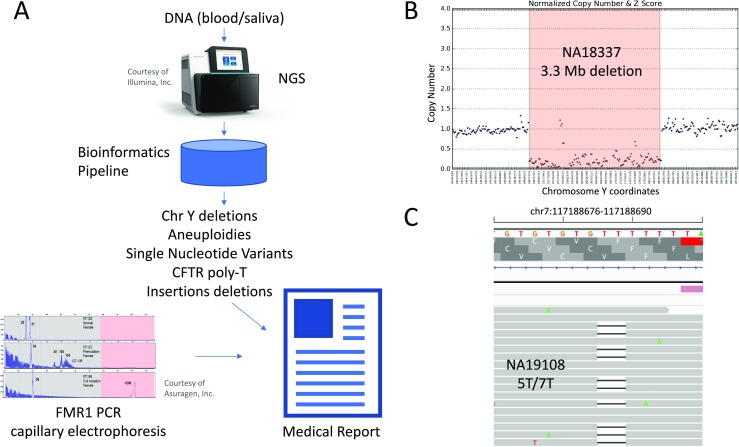
**a** Outline of our NGS test. A DNA sample (saliva or blood) is sequenced by NGS and processed by our custom bioinformatics pipeline. Y chromosome microdeletions, sex chromosome aneuploidies, *CFTR* IVS8-5T polymorphism, indels, and SNVs are called. Variants are interpreted by expert curators and a medical report is generated. In parallel, *FMR1* testing is performed using PCR and capillary electrophoresis, and results are incorporated into the medical report. **b** Example of Y chromosome microdeletion called in sample NA18337. **c** Example of IVS8-5T tract detection in sample NA19108, heterozygous for 5T/7T

To maximize the clinical impact of our comprehensive infertility genetic test, we focused only on genes with demonstrated impact on infertility phenotype. Genes were classified as “diagnostic” when variants in them were reported to cause infertility across multiple populations, as supported by multiple publications from different laboratories, demonstrating a direct relationship with infertility. Genes were classified as “informative” when variants in them were reported to be associated with infertility, but the causality link has not been unequivocally established. For male infertility, the panel includes Y chromosome microdeletions, *CFTR* mutations, and sex chromosome aneuploidies [6]. For female infertility, the panel includes sex chromosome aneuploidies and genes in which variants have been associated with recurrent pregnancy loss caused by thrombophilia, primary ovarian insufficiency, polycystic ovary syndrome, and ovarian hyperstimulation syndrome. The gene list, as well as the rationale used for their selection, is shown in Fig. 1 and Supplementary Table S2. Figure 2 describes the laboratory and analysis workflow.

### Analytical and clinical validation

To validate panel performance and determine analytical sensitivity, specificity, and accuracy, we sequenced 24 samples from the 1000 Genomes (1000G) project [20] for which the location of SNVs and indels are known (Supplementary Table S1). Representative sequencing quality control statistics are shown in Supplementary Table S4. NGS results from these validation samples were compared to known 1000G variants (Table 1). Microarray analysis and/or Sanger sequencing was performed to assess the discrepancies of SNVs/indels between results produced by the NGS panel and previously known 1000G data. Analytical sensitivity of the test was > 99% for SNVs and > 91% for indels, and specificity was > 99% for both SNVs and indels. Final accuracy for SNVs and indels was 99.98 and 99.42%, respectively.

**Table 1:** Performance characteristics of the NGS test. *PPV*, positive predictive value; *NPV*, negative predictive value; *CNV*, copy number variant

Variant type	True positives	True negatives	False positives	False negatives	
snp	4578	257,918	2	40	
Indel	137	2106	0	13	
Variant type	Accuracy	Sensitivity	Specificity	PPV	NPV
snp	99.98%	99.13%	99.98%	99.96%	99.98%
Indel	99.42%	91.33%	99.39%	100.00%	99.39%
Batch type	Concordant	Discordant	Concordance		
Intra-batch	588	17	97.19%		
Inter-batch	427	28	93.85%		

To determine clinical sensitivity in the detection of CNVs, sex chromosome aneuploidies and Y chromosome microdeletions, we used 34 samples with 38 known variants (Supplementary Table S1). The set of variants previously known for these samples is limited, so specificity could not be assessed. The analysis correctly detected 3/3 CNVs, 19/19 sex chromosome aneuploidies, and 15/16 Y chromosome microdeletions (Supplementary Tables S5 and S6, Table 1 and Fig. 2). In three cases (NA20435, NA18333, and NA22031), Y chromosome microdeletions of smaller size were found by NGS when compared to previously known data (Supplementary Table S6); however, microarray analysis confirmed the previously reported microdeletion size. In one case (NA20434), NGS data located the microdeletion 1.17 Mbp downstream of the previously known location, and microarray analysis confirmed NGS result. Sample NA12662 has a sex chromosome aneuploidy (duplication of X chromosome) and a Y chromosome microdeletion ((22769319-27097245)x0). The sex chromosome aneuploidy was identified, but the Y chromosome microdeletion was missed. Thus, clinical sensitivity for sex chromosome aneuploidies and CNVs is 100% (22/22), and for Y chromosome microdeletions, it is 93.75% (15/16).

Next, 11 DNA samples with 17 known SNVs/indels were processed (Table 2). Known variants were confirmed in 16/17 cases. Sample NA02795 was reported with a pathogenic variant *GALT* c.130G>A; p.Val44Met. However, this variant was not identified by NGS and Sanger sequencing did not detect it either; therefore, based on NGS and Sanger sequencing concordance, we did not count this case against the sensitivity. Thus, clinical sensitivity for SNVs/indels by our assay was 100%.

**Table 2:** Clinical sensitivity was determined by sequencing SNVs/indels of known variants

Sample	Previously reported	NGS test call
NA11763	*GALT* c.591A>G; p.Gln188Arg	*GALT* NM_000155.3:c.563A>G; p.Gln188Arg
NA11763	*GALT* c.1025C>T; p.Arg333Trp	*GALT* NM_000155.3:c.997C>T; p.Arg333Trp
NA14899	*F5* c.1691G>A; p.Arg506Gln (homozygous)	*F5* NM_000130.4:c.1691G>A; p.Arg506Gln
IndianaBiobank02	*SERPINC1* c.1148_1149delTC; p.Leu383Profs*10	*SERPINC1* NM_000488.3:c.1148_1149delTC; p.Leu383Profs*10
NA00422	*GALT* c.563A>G; p.Gln188Arg	*GALT* NM_000155.3:c.563A>G; p.Gln188Arg
NA00422	*GALT* c.1030C>A; p.Gln344Lys	*GALT* NM_000155.3:c.1030C>A; p.Gln344Lys
NA02795	*GALT* c.425T>A; p.Met142Lys	*GALT* NM_000155.3:c.425T>A; p.Met142Lys
NA02795	*GALT* c.130G>A; p.Val44Met	None
DNAsimple01	*CFTR* c.1584G>A; p.Glu528=	*CFTR* NM_000492.3:c.1584G>A; p.Glu528=
NA02796	*GALT* c.512T>C; p.Phe171Ser	*GALT* NM_000155.3:c.512T>C; p.Phe171Ser
NA02796	*GALT* c.404C>T; p.Ser135Leu	*GALT* NM_000155.3:c.404C>T; p.Ser135Leu
NA16643*	*F5* c.1691G>A; p.Arg506Gln	*F5* NM_000130.4:c.1601G>A; p.Arg534Gln
NA17431	*GALT* c.591A>G; p.Gln188Arg	*GALT* NM_000155.3:c.563A>G; p.Gln188Arg
NA17431	*GALT* c.612T>C; p.Leu195Pro	*GALT* NM_000155.3:c.584T>C; p.Leu195Pro
NA16000	*MTHFR* c.677C>T; p.Ala222Val	*MTHFR* NM_005957.1:c.677C>T; p.Ala222Val
NA16000	*F2* c.20210G>A (homozygous)	*F2* NM_000506.4:c.*97G>A
DNAsimple02	*F2* c.20210G>A	*F2* NM_000506.4:c.*97G>A

*The official designation of F5 c.1691G>A is NM_000130.4:c.1601G>A; NP_000121.2:p.Arg534Gln

### CFTR intron 8 poly dT (IVS8-5T)

Varied lengths of a thymidine (T)-tract (5, 7, or 9T) are found in front of the splice-acceptor site of intron 8 of the *CFTR* gene in males with congenital bilateral absence of the vas deferens [21]. The length correlates with the efficiency of exon 9 splicing. This polymorphism is detected clinically by allele-specific multiplex PCR [22]. In order to determine sensitivity of the NGS panel, we analyzed 72 samples from the 1000G project. When 1000G data was used as reference, the NGS panel correctly detected the IVS8 alleles in 67/72 cases, and for the 5 discrepant cases, Sanger sequencing demonstrated that the NGS panel call was the correct one (Supplementary Table S7). Therefore, our assay had 100% sensitivity to detect the *CFTR* IVS8-5T allele.

### FMR1 testing

CGG triplet expansions are known to cause fragile X syndrome, and alleles in the pre-mutation range have been associated with increased risk for premature ovarian failure [23]. Current NGS protocols based on hybridization enrichment for targeted panels do not accurately quantify the number of repeats. Therefore, to complement the NGS panel with *FMR1* analysis, we used an already available protocol based on PCR amplification and capillary electrophoresis detection of CGG repeats [24]. Test sensitivity was determined by analysis of 26 samples harboring different CGG repeat expansions (Supplementary Table S8). We correctly identified all alleles previously classified as “mutation” (> 200 CGG repeats), “pre-mutation” (55 to 199 CGG repeats), and “normal” (< 45 CGG repeats). However, the assay misclassified one allele in the “intermediate” range (between 45 and 54 CGG repeats) as “pre-mutation” (NA20236, 53 vs 55 CGG repeats, Supplementary Table S8). Given that the rate of expansions of intermediate alleles is not well understood, testing at-risk relatives of individuals with an intermediate allele may determine the stability of the allele in the family [25].

### Cost analysis

Currently, after semen analysis, in cases where severe abnormal semen analysis is observed, genetic testing for variants associated with male infertility is performed with multiple assays. For example, *CFTR* IVS8-5T is detected by allele-specific multiplex PCR [22], Y chromosome microdeletions are detected by PCR of sequence-tagged sites [26], and sex chromosome aneuploidies are detected by traditional karyotyping or microarrays [27]. NGS can detect all these variants using a single test. In order to determine the economic impact, we performed a cost analysis for the male fertility panel, using average pricing available from reference laboratories performing these assays (Table 3 and Supplementary Table S9). Overall, the savings to providers could be as much as $2723 per case, $3322 if performed by multiple assays vs $599 if performed by NGS, which translates in savings of > 550%. Furthermore, NGS testing has a shorter turnaround time because there is no need to do reflex testing. The only exception may be cases with complex chromosomal rearrangements and mosaics or translocations/inversions that do not result in gain/loss of DNA and are, therefore, not captured by the NGS panel.

**Table 3:** Cost comparison between traditional diagnostic methods for male infertility and NGS diagnostic test

Variant	Methodology	CPT code	Cost ($US)
CFTR IVS8 poly T tract	Multiplex polymerase chain reaction, Sanger sequencing or NGS	81,224	396
CFTR mutations	Sanger sequencing of CFTR gene	81,223	1430
Sex chromosome aneuploidies	Karyotype	88,262	946
Y chromosome microdeletion	PCR-based analysis of 14 different regions along the length of the Y chromosome	81,403	660
		Total cost:	3322
NGS infertility test	NGS sequencing, includes confirmation with orthogonal methods (microarray or Sanger)	81,224, 81,223, 88,262, 81,403	599

## Discussion

We present a comprehensive genetic test based on NGS that covers the main infertility indications [6, 12]: the female panel analyzes genes associated with increased risks for female infertility, including primary ovarian insufficiency, polycystic ovary syndrome, sex chromosome aneuploidy, ovarian hyperstimulation syndrome, and thrombophilia-related pregnancy loss (Fig. 1). The male panel includes Y chromosome microdeletions, congenital absence of the vas deferens, sex chromosome aneuploidy, and other causes of male factor infertility (Fig. 1).

From a clinical care perspective, this NGS test has the ability to influence key decisions in patient management following infertility diagnosis. In the case of female infertility patients, a complement or earlier diagnosis of predisposition to severe diminished ovarian response or premature ovarian failure may help providers to better guide patients to gamete cryopreservation in anticipation of future pregnancies. For patients found to have a definitive diagnosis of polycystic ovary syndrome, it has been shown that patients who receive a gonadotropin-releasing hormone antagonist protocol undergoing controlled ovarian stimulation experience reduced risk of ovarian hyperstimulation syndrome [28].

For male infertility patients, it is a routine for IVF laboratories or reproductive urologists to perform semen analysis for sperm count, motility, and morphology. For those men with results indicative of severe oligospermia or azoospermia, it is a routine to reflex to genetic tests to help explain etiology and guide treatment. Depending on the nature of the mutations, providers are able to better determine whether male patients are candidates for surgical retrieval of sperm [29]. Reduced cost and faster turnaround time in genetic analysis will help patients reach the proper treatment more efficiently.

Genetic analysis is currently performed using many different platforms: SNVs and indels are detected by Sanger sequencing or NGS, sex chromosome aneuploidies by karyotyping or microarrays, Y chromosome microdeletions by multiplex PCR of sequence tags, and *CFTR* intron 8 polymorphism by allele-specific multiplex PCR. NGS allows for the detection of these different variants using a single platform with excellent sensitivity and specificity. Out of 127 different events, the NGS panel complemented with orthogonal confirmation methods accurately detected almost all of them: 17/17 SNVs/indels, 3/3 CNVs, 19/19 sex chromosome aneuploidies, 15/16 Y chromosome microdeletions, and 72/72 *CFTR* IVS8 polymorphic sites (5 of them IVS8-5T). In three Y chromosome microdeletion cases, the NGS panel identified microdeletions of smaller size; however, an analysis using microarrays confirmed the previously known size in all three cases. The NGS panel, thus, identified accurately most Y chromosome microdeletions (one Y chromosome microdeletion was missed), but orthogonal methods are required to confirm the microdeletion size. In summary, overall clinical sensitivity for all variants is 99.21% (126/127).

Our study has several limitations. Sample size is limited, and even though we processed samples representing all types of variants, the final number of samples in each group is small. For example, we only processed three clinical samples containing CNVs. Therefore, given limited sample size, performance characteristics must be interpreted with caution. In addition, this is a retrospective study, so it serves as a proof of concept, but a prospective study (currently ongoing at our center) will be needed to confirm our findings.

In its current form, the NGS panel cannot detect balanced translocations; therefore, it would miss reciprocal translocations and Robertsonian translocations, which are known to cause infertility in 0.9% of men [9]. Furthermore, the NGS panel cannot detect complex chromosomal rearrangements like derivative chromosomes or mosaicism, even though these often involve gain or loss of DNA. We tested several samples with derivative chromosomes and mosaicism and the current NGS panel could not accurately assess these variants. For example, samples IndianaBiobank01 and NA21681 in Supplementary Table S5 contain a derivative X,Y chromosome and a 6,Y translocation, respectively, and both were missed. In these cases, a karyotype is needed, which could be used as reflex after the NGS test. We are currently investigating the possibility to incorporate detection of these abnormalities as part of this NGS test [30].

Another limitation is the detection of *FMR1* variant caused by triplet CGG expansion, varying from 20 to > 900 repeats. It is not possible to accurately identify triplet expansions by using PCR-enriched NGS. New methods involving PCR-free libraries allow diagnosis [31], but it would require a parallel library preparation and sequencing reaction, significantly increasing cost. Therefore, currently, a separate PCR assay to determine *FMR1* mutation is needed to assess fragile-X mutation.

The direct consequence of using a single NGS assay instead of multiple diagnostic assays, each one detecting a class of DNA variants, is a potential reduction in cost and turnaround time required to make a definitive diagnosis, with savings that could be up to $2723 per case ($3322 for traditional methods vs $599 for NGS testing). Integrating testing into a single assay simplifies test ordering and result tracking for the clinician and decreases cost to the patient by reducing the number of assays that need to be performed, analyzed, and reported.

## Conclusions

We have developed a comprehensive genetic test for female and male infertility that achieves excellent clinical sensitivity, but at a fraction of the cost of traditional methods of testing, and a shorter turnaround time than current methods.

## Electronic supplementary material

ESM 1 (XLSX 208 kb)

ESM 2 (DOCX 130 kb)
