# Supplementary material for: Comprehensive genetic testing for female and male infertility using next-generation sequencing
Source: J Assist Reprod Genet. 2018 May 19;35(8):1489–96. doi: 10.1007/s10815-018-1204-7 (PMC6086787; doi:10.1007/s10815-018-1204-7)
Supplement: Supplementary file 2 — (DOCX 130 kb) [file 10815_2018_1204_MOESM2_ESM.docx]

**Supplementary Methods**

*Next Generation DNA Sequencing*

DNA samples were prepared for sequencing using HyperPlus Library Preparation Kit (Roche, Indianapolis, IN). Following the manufacturer’s instructions, 250 ng of each genomic DNA sample were fragmented for 20 minutes, end repaired, A-tailed, and ligated with 48 adaptors (BIOO Scientific, Austin, TX), and then size selected using AMPure beads (Beckman Coulter, Indianapolis, IN) for fragments averaging 350 bp in length. DNA was amplified and after confirming DNA concentration and integrity with a LabChip Gx Touch II (Perkin Elmer, Waltham, MA), 1ug total DNA was hybridized with a SeqCap EZ oligo pool (Roche), following the manufacturer’s instructions. The appropriate blockers were included from BIOO Scientific’s 48 Blockers following the SeqCap EZ Protocol and the hybridization was incubated for 16 hours at 47°C. The SeqCap EZ system (Roche) was used for DNA hybridization and enrichment. The DNA libraries were diluted to 4nM and the enriched DNA integrity and concentration was checked using NGS library Quantification kit for Illumina libraries (Roche). Libraries were sequenced on a NextSeq500 (Illumina, San Diego, CA) using a High Output 300 cycle reagent kit following the manufacturer’s instructions.

*Bioinformatics pipeline*

All bioinformatics algorithms were implemented within the Elements^TM^ platform (Phosphorus, New York, NY). FASTQ files were produced from each sequencing run and processed using the germline calling pipeline (version 2.03.01.30066) in DRAGEN (Edico Genome, San Diego, CA). Variants identified by NGS were confirmed by an orthogonal method (microarrays or Sanger sequencing). After confirmation, each variant was classified as pathogenic, likely pathogenic, variant of unknown significance (VUS) or benign, following American College of Medical Genetics (ACMG) guidelines (1).

CNV identification was performed together with Y chromosome microdeletion detection. From all regions captured by the panel (entire BED file), we removed unmappable regions using data from GA4GH (https://github.com/ga4gh, human_g1k_v37_gemmap_l250_m2_e1_nonuniq.bed), to create a BED file without repeat regions. NGS sequencing data was processed by binning intervals into 100bp length fragments, and removing intervals with extreme GC contents (>80% or <20%). We then computed average depth of each interval using read depth given by the DRAGEN (Edico Genome, CA) pipeline (see DRAGEN manual for details) and removed intervals with low depth (<20x) or zero coverage across samples. We also removed samples with low Pearson correlation coefficients compared to other samples in the same batch. We adopted spline normalization to normalize interval depth and removed intervals with extreme high depth (>0.1%) as well as high variable (>0.1%). Next, we removed top principal components (PCA analysis) to control batch effects. We adopted the Circular Binary Segmentation algorithm 2(2) to find breakpoints as boundaries of CNV. In general, we defined mean copy number (each interval depth divided by median depth of that interval across all samples) > 2.5 as duplication and < 1.5 as deletion. The significance of regions called is tested using permutation on z-scores of normalized interval depth. Last, we focused on > 5million bp regions so as to further narrow down to crucial genes and reduce false positives.

The algorithm to call Y chromosome microdeletions is the same as for autosome CNV calling, except that we included Y chromosome regions with low mappability but good coverage.

For aneuploidy detection, we calculated ratio of Y chromosome or X chromosome signal to the mean of autosomes, and rounded to integer. We defined ploidy as below (autosomes are diploid, so the ratio is half of the ploidy class):

| Ploidy class | X ratio | Y ratio |
| --- | --- | --- |
| 0 | ≤ 0.07 | ≤ 0.07 |
| Between 0 and 1 | 0.07-0.45 | 0.07-0.35 |
| 1 | 0.45-0.53 | 0.35-0.51 |
| Between 1 and 2 | 0.53-0.87 | 0.51-0.78 |
| 2 | 0.87-1.03 | 0.78-0.96 |
| Between 2 and 3 | 1.03-1.39 | 0.96-1.21 |
| 3 | 1.39-1.51 | 1.21-1.37 |
| 3 or more | ≥ 1.51 | ≥ 1.37 |

*Microarrays*

A custom Affymetrix axiom array was designed based on Affymetrix myDesign requirements. The array includes quality control (QC) SNVs, variants and CNVs probesets. QC SNP probesets are used to optimize and standardize genotyping and sample QC and are comprised of a set of 1136 SNVs used to assess genetic ancestry (3), 9771 SNVs to assess identity-by-descent (4) and 90 individual identification SNVs (5). A set of 110K pathogenic and likely pathogenic variants were extracted from ClinVar database and are represented by at least one probeset in the array. Finally, 517K probesets spanned all exons in the panel and the sex chromosomes, and were used for CNV and aneuploidy detection.

DNA samples (200 ng) were hybridized using the Axiom 2.0 Assay method (Affymetrix, Santa Clara, CA) following the manufacturer’s instructions for denaturation, neutralization, DNA amplification and hybridization. DNA integrity and concentration was confirmed with a LabChip Gx Touch II (Perkin Elmer). Microarrays were analyzed in a GeneTitan (Affymetrix) scanner.

Raw data were processed using Affymetrix Power Tools v1.20.5. For a sample to pass quality control, it required a DishQC (DQC) score ≥0.82 and a first pass sample call rate (CR) ≥97%. Genotyping quality analysis for each probe set was performed using SNPolisher R package (Affymetrix) and in-house scripts (available at XXXXX). Probe sets with a call rate <95%, Fisher’s linear discriminant <3.6, heterozygous strength offset <-0.1, homozygote ratio offset <0.5, mean of AA <0.01, mean of BB> 0, distance of AB> 1.58, or distance between homozygous cluster AA and BB <1.52 were filtered out.

A set of 1598 probesets that pass the SNP QC metrics with Minor Allele Frequency (MAF) <0.005% in 1000 Genome project samples were selected as the indicator probe sets. These 1598 probesets were used to normalize each CNV target region. For copy number variants detection, a custom script that implements RANSAC linear regression models was used to calculate the observed R ratio and predicted R ratio for each exon and assess the copy number level. R value is calculated as [Log2(A_intensity) + Log2(B_intensity)]/2. R ratio is calculated by dividing sample’s R value by the median R value of that probeset for all samples.

*Sanger Sequencing*

DNA to be sequenced was first amplified by polymerase chain reaction (PCR). Each PCR reaction contained 50 ng of genomic DNA, 2.5 uL of each primer (2 uM) and 12.5 uL of Platinum Multiplex PCR MasterMix (Thermo Scientific, Waltham, MA) in a final volume of 25 ul. Thermal cycling was performed using a Veriti 96-well thermal cycler (Applied Biosystems, Foster City, CA) with the following conditions: 95°C for 2 minutes, followed by 35 cycles of 95°C for 30 seconds, 60°C for 1 minute and 30 seconds, and 72°C for 1 minute, followed by a final extension at 72°C for 10 minutes. PCR product was cleaned using AMPure XP (Beckman Coulter, Indianapolis, IN) following the manufacturer’s instructions. The PCR was then quantified using Quant-iT PicoGreen and diluted to 5 ng/uL. 1 uL was used to perform BigDye Sanger sequencing on a 3500XL capillary electrophoresis (Applied Biosystems). Analysis was performed using the Elements^TM^ platform (Phosphorus).

References

1. Richards S, Aziz N, Bale S, Bick D, Das S, Gastier-Foster J, et al. Standards and guidelines for the interpretation of sequence variants: a joint consensus recommendation of the American College of Medical Genetics and Genomics and the Association for Molecular Pathology. Genet Med [Internet] 2015;17(5):405–23. Available from: http://dx.doi.org/10.1038/gim.2015.30

2. Olshen AB, Venkatraman ES, Lucito R, Wigler M. Circular binary segmentation for the analysis of array-based DNA copy number data. Biostatistics 2004;5(4):557–72.

3. Shraga R, Yarnall S, Elango S, Manoharan A, Rodriguez SA, Bristow SL, et al. Evaluating genetic ancestry and self-reported ethnicity in the context of carrier screening. BMC Genet 2017;18(1):1–9.

4. Thompson EA. Identity by descent: Variation in meiosis, across genomes, and in populations. Genetics 2013;194(2):301–26.

5. Pakstis AJ, Speed WC, Fang R, Hyland FCL, Furtado MR, Kidd JR, et al. SNPs for a universal individual identification panel. Hum Genet 2010;127(3):315–24.
